# HLA high-resolution typing by next-generation sequencing in Pandemrix-induced narcolepsy

**DOI:** 10.1371/journal.pone.0222882

**Published:** 2019-10-02

**Authors:** Alexander Lind, Omar Akel, Madeleine Wallenius, Anita Ramelius, Marlena Maziarz, Lue Ping Zhao, Daniel E. Geraghty, Lars Palm, Åke Lernmark, Helena Elding Larsson

**Affiliations:** 1 Department of Clinical Sciences Malmö, Lund University/CRC, Skåne University Hospital SUS, Malmö, Sweden; 2 Clinical Research Division, Fred Hutchinson Cancer Research Center, Seattle, Washington, United States of America; 3 Section for Paediatric Neurology, Department of Paediatrics, Skåne University Hospital SUS, Malmö, Sweden; Charite Medical University Berlin, GERMANY

## Abstract

The incidence of narcolepsy type 1 (NT1) increased in Sweden following the 2009–2010 mass-vaccination with the influenza Pandemrix-vaccine. NT1 has been associated with Human leukocyte antigen (*HLA*) DQB1*06:*02* but full high-resolution HLA-typing of all loci in vaccine-induced NT1 remains to be done. Therefore, here we performed HLA typing by sequencing *HLA*-*DRB3*, *DRB4*, *DRB5*, *DRB1*, *DQA1*, *DQB1*, *DPA1* and *DPB1* in 31 vaccine-associated NT1 patients and 66 of their first-degree relatives (FDR), and compared these data to 636 Swedish general population controls (GP). Previously reported disease-related alleles in the *HLA*-DRB5*01:*01*:01-DRB1*15:*01*:01-DQA1*01:*02*:01-DQB1*06:*02*:*01* **extended haplotype** were increased in NT1 patients (34/62 haplotypes, 54.8%) compared to GP (194/1272 haplotypes, 15.3%, *p* = 6.17E^-16^). Indeed, this extended haplotype was found in 30/31 patients (96.8%) and 178/636 GP (28.0%). In total, 15 alleles, four extended haplotypes, and six genotypes were found to be increased or decreased in frequency among NT1 patients compared to GP. Among subjects with the *HLA*-DRB5*01:*01*:01-DRB1*15:*01*:01-DQA1*01:02-DQB1*06:*02* haplotype, a second DRB4*01:*03*:01-DRB1*04:*01*:01-DQA1*03:02//*03:*03*:01-DQB1*03:*01*:*01* haplotype (*p* = 2.02E^-2^), but not homozygosity for DRB1*15:*01*:01-DQB1*06:*02*:*01* (*p* = 7.49E^-1^) conferred association to NT1. Alleles with increased frequency in DQA1*01:*02*:*01* (p = 1.07E^-2^) and DQA1*03:02//*03:*03*:*01* (p = 3.26E^-2^), as well as with decreased frequency in DRB3*01:*01*:*02* (p = 8.09E^-3^), DRB1*03:*01*:*01* (*p* = 1.40E^-2^), and DQB1*02:*01*:*01* (*p* = 1.40E^-2^) were found among patients compared to their FDR. High-resolution *HLA* sequencing in Pandemrix-associated NT1 confirmed the strong association with the DQB1*06:*02*:*01*-containing haplotype but also revealed an increased association to the not previously reported extended *HLA*-DRB4*01:*03*:01-DRB1*04:*01*:01-DQA1*03:02//*03:*03*:01-DQB1*03:*01*:*01* haplotype. High-resolution *HLA* typing should prove useful in dissecting the immunological mechanisms of vaccination-associated NT1.

## Introduction

Narcolepsy type 1 (NT1) is a chronic disease characterized by excessive daytime sleepiness and disturbed nocturnal sleep [1, 2]. The underlying cause behind NT1 is a specific degeneration of the hypocretin producing neurons in the lateral hypothalamus [3, 4]. The cell loss, together with the near-complete association to Human leukocyte antigen (HLA) DQB1*06:*02* [5], suggests an autoimmune etiology. Historically, it has been difficult to fulfill criteria for autoimmunity in narcolepsy through the identification of autoantigens or disease-specific autoantibodies [6] as well as a disease-related appearance of hypothalamic immune-cell infiltration [2]. Recent studies have identified autoreactive T cells against hypocretin neurons and peptides in NT1 [7–9]. The HLA-DQB1*06:*02* allele is present in about 20% of the general European population; it is more common in northern than in southern countries [5]. Consequently, *HLA*-DQB1*06:*02* has been considered as a necessary but not sufficient genetic factor for NT1, since this allele is common in the population, and the incidence of the disease is low. In *HLA*-DQA1*01:02-DQB1*06:*02* heterozygous individuals, *HLA*-DQB1^*^03:*01* was linked to spontaneous NT1 as the second allele. In contrast, *HLA*-DQA1^*^01:01-DQB1^*^05:*01*, DQA1^*^01:03-DQB1^*^06:*03*, and DQA1^*^01:03-DQB1^*^06:*01* were decreased in frequency among NT1-patients as the second haplotype to DQA1*01:02-DQB1*06:*02* [5, 10–15].

The Pandemrix-vaccination in Sweden and Finland following the 2009 influenza pandemic resulted in an up to 15-fold increased incidence of NT1 in both countries [16–21]. Moreover, an increased incidence of NT1 was reported in China following the 2009 influenza pandemic [22, 23]. Follow-up studies suggest differences between pre- and post-2009 pandemic NT1 patients in their underlying *HLA* genetics, decreased frequency of *HLA*-DQB1*06:*02* homozygosity and *HLA*-DQB1*03:*01* associated with a young age at onset [10]. *HLA*-*DQB1* allelic variants associated with NT1 appear comparable between Pandemrix-induced and spontaneous disease [24], but the full *HLA* genetics for vaccine-induced NT1 remains to be determined. It is currently not known whether Pandemrix-associated NT1 share all *HLA* characteristics with spontaneous NT1. To address this question, this study aimed to determine *HLA*-*DRB3*, *DRB4*, *DRB5*, *DRB1*, *DQA1*, *DQB1*, *DPA1*, and *DPB1* by high-resolution sequencing [25–27] in 31 Pandemrix-associated NT1 patients, 66 first-degree relatives (FDR), and 636 Swedish general population controls (GP).

## Materials and methods

### Study populations

In collaboration with the Swedish Narcolepsy Association, families with one member affected by Pandemrix-induced NT1 were informed about the study and gave consent to participate. The legally authorized representatives, parents or guardians of under aged study participants gave informed consent. In total, 31 patients and 66 FDR, geographically scattered all over Sweden, agreed to donate blood samples at their local Health Center. To the best of our knowledge, the patients had cataplexies and were treated accordingly.

The Swedish population controls (n = 448) subjected to high-resolution HLA typing were reported previously [27] and added to LifeGene (https://www.lifegene.se/For-scientists/About-LifeGene/) population controls (n = 188), representing the control group (GP, n = 636) in the present study. The LifeGene controls were sequenced simultaneously with the NT1 families. The present study is part of the AMINA-Study (Autoimmune Multiple Sclerosis and Narcolepsy), a Swedish research project focused on neurological autoimmune diseases. The study was approved by the Regional Ethical Review Board in Lund, Sweden (dnr 2015/257). All study participants gave written consent to participate.

### DNA extraction

DNA from all participants (n = 97) in the NT1 families were extracted using a QIAmp Blood Maxi Kit (Qiagen) according to the manufacturer’s instructions. Two 8 mL CPT Mononuclear Cell Preparation Tubes (ref 362 761, Becton Dickinson, Franklin Lakes, New Jersey, U.S.) of whole blood were collected, and DNA was extracted from the remaining erythrocyte layer in the tubes following Peripheral Blood Mononuclear cell (PBMC) isolation. CPT tubes were centrifuged for 5 min at 2000 rpm to free the erythrocyte layer underneath the gel plug. The blood clots were lysed in 0.5 mL Qiagen protease and 6 mL DNA purification AL lysis buffer according to the manufacturer’s instructions. Incubation for 10 min in a 70°C water-bath was followed by the addition of 5 mL 99.5% ethanol. The reaction mixture was applied into QIAamp maxi columns; DNA was bound to the filter during 3 min centrifugation at 4000 rpm. The eluate was discarded and the columns were washed in 5 mL washing buffers AW1 followed by AW2. DNA was eluted in two steps by the addition of 2 × 600 μL Buffer AE and later pooled. The DNA concentration was measured using a Nanodrop device (Thermo Fisher Scientific, Waltham, MA, USA). All samples were diluted in nuclease-free water to a concentration of 50 ng/μL DNA.

### *HLA* high-resolution sequencing

*HLA* Class II -*DRB3*, *DRB4*, *DRB5*, *-DRB1*, *-DQA1*, *-DQB1*, *-DPA1*, and *-DPB1* typing was carried out using the ScisGo *HLA* v4 typing kit (Scisco Genetics Inc., Seattle, WA). Briefly, the method employs an amplicon-based 2-stage PCR, followed by sample pooling and sequencing using a MiSeq v2 PE500 (Illumina, San Diego, CA) [25–27]. The inheritance by descent within the NT1 families allowed us to decipher the extended *HLA*-*DRB3*, *DRB4*, *DRB5*, *DRB1*, *DQA1*, *DQB1*, *DPA1*, and *DPB1* haplotypes. The extended *HLA* haplotypes for GP were assembled based on the haplotypes identified by descent in the NT1 families. The process to assemble the extended haplotypes of the GP subjects were performed as follows (S1 Table);

1. Homozygous individuals were identified.
2. Individuals heterozygous for *HLA*-DRB5*01:*01*:01-DRB1*15:*01*:01-DQA1*01:*02*:01-DQB1*06:*02*:*01* were identified. These alleles were assigned to the first haplotype; the remaining alleles were assigned to the second haplotype.
3. Step 2 revealed additional extended haplotypes among individuals heterozygous for *HLA*-DRB5*01:*01*:01-DRB1*15:*01*:01-DQA1*01:*02*:01-DQB1*06:*02*:*01*. A total of sixteen extended haplotype variants were identified.
4. A total of 634/636 control subjects could be assigned to two extended haplotypes according to the scheme in step 3 (S1 Table). Conflicting results were found for two individuals; in both cases, the two extended haplotypes were matched to the same *HLA*-*DRB3*, *DRB4*, *DRB5* allele and not matched to the other. This was solved by assigning the matched *HLA*-*DRB3*, *DRB4*, and *DRB5* alleles to the more common haplotype.

The Swedish populations’ controls (n = 448) [27] and LifeGene controls (n = 188) were sequenced at two separate occasions with improved typing resolution the second time. As a consequence, DQA1*03:*02* for the former group could be separated into DQA1*03:*02*:*01* and DQA1*03:*03*:*01* in the latter group. To be able to merge the groups and compare the controls to NT1-families (typed at the same time as LifeGene controls), the DQA1*03:*02* allele was considered as one common DQA1*03:02//*03:*02*:01//*03:*03*:*01* allele. To simplify the reading of the manuscript, the group was abbreviated to DQA1*03:02//*03:*03*:*01*.

### Statistical analyses

#### RPEs

Relative predispositional effect analysis was performed as previously described [28]. The analysis was performed with the χ2 test or Fisher's exact test using R-studios and the package “epiDisplay” (https://cran.r-project.org/web/packages/epiDisplay/epiDisplay.pdf).

In brief, the allele with the largest deviation between groups was identified and excluded. This sequential elimination of most significant effect was repeated in a new round of calculations. The procedure was redone until no further alleles with a significant deviation between the compared groups could be identified. For this analysis, alleles with a low number of narcolepsy-patients (≤ 1) were not considered significant.

#### Haplo.score

Calculations were performed using R-studios and the function “haplo.cc” (http://cran.r-project.org/web/packages/haplo.stats/index.html), as previously described [27, 29, 30].

In brief, allelic frequencies were compared between NT1 patients to GP or FDR. Allelic/haplotypic associations were assessed using Haplotype-score test. Odds ratios were computed following selection of a reference allele/haplotype with a comparable frequency in both patients and controls. The odds ratio for the reference allele/haplotype was 1, higher and lower values being associated with increased and decreased risk, respectively.

## Results

The present analysis represents next-generation high-resolution sequencing extended to both exons 2 and 3 for all *HLA*-*DR*, *DQ*, and *DP* alleles. The analyses follow the allele location on chromosome 6, and allele frequencies are followed by haplotypes, extend haplotypes and genotypes.

### *HLA*-*DRB3*, *DRB4*, *DRB5* alleles

The typing of *HLA*-*DRB3*, *DRB4*, and *DRB5* revealed ten alleles with a frequency of at least 1% among NT1 patients or general population controls (GP) (S2 Table). A total of seven *HLA*-*DRB3*, *DRB4*, and *DRB5* alleles were found only among GP and with a frequency below 1% (S3 Table). Relative predispositional effects (RPEs) showed that DRB5*01:*01*:*01*, DRB4*01:*03*:*01* and DRB3*03:*01*:*01* were associated to NT1 compared to GP (Table 1 and S4 Table). HLA-DRB3*01:*01*:*02* were found at a lower frequency among NT1 patients than among FDRs (Table 1 and S5 Table).

**Table 1:** RPE analysis of *HLA-DRB3*, *DRB4*, *DRB5*, *DRB1*, *DQA1*, *DQB1*, *DPA1*, and *DPB1* allele frequencies among NT1 patients (n = 31), population controls (n = 636) and first-degree relatives (FDR, n = 66).

Allele	Controls n (%)	NT1 n (%)	OR (95% CI)	p-value	p-value	p-value
DRB3*03:*01*:*01*	56 (4.4%)	4 (6.5%)	1.5 (0.38, 4.26)	3.57E-01	6.21E-02	2.32E-02
DRB4*01:*03*:*01*	307 (24.1%)	13 (21%)	0.83 (0.45, 1.56)	5.68E-01	4.26E-02	
DRB5*01:*01*:*01*	205 (16.1%)	34 (54.8%)	6.32 (3.75, 10.65)	8.24E-15		
DRB1*04:*01*:*01*	126 (9.9%)	7 (11.3%)	1.16 (0.52, 2.6)	7.22E-01	3.42E-02	
DRB1*13:*02*:*01*	56 (4.4%)	4 (6.5%)	1.5 (0.38, 4.26)	3.57E-01	6.16E-02	3.64E-02
DRB1*15:*01*:*01*	202 (15.9%)	34 (54.8%)	6.43 (3.82, 10.84)	4.17E-15		
DQA1*01:*02*:*01*	265 (20.8%)	38 (61.3%)	6.02 (3.55, 10.21)	1.13E-13		
DQA1*03:02//*03:*03*:*01*	81 (6.4%)	9 (14.5%)	2.5 (1.19, 5.24)	1.25E-02	4.36E-07	
DQB1*03:*01*:*01*	203 (16%)	11 (17.7%)	1.14 (0.58, 2.22)	7.09E-01	7.32E-03	
DQB1*06:*02*:*01*	201 (15.8%)	34 (54.8%)	6.47 (3.84, 10.91)	3.31E-15		
DQB1*06:*04*:*01*	52 (4.1%)	4 (6.5%)	1.62 (0.41, 4.63)	3.26E-01	4.97E-02	1.84E-02
DPA1*02:*06*	6 (0.5%)	3 (4.8%)	10.68 (1.69, 51.4)	6.58E-03		
DPB1*02:*01*:*02*	178 (14%)	3 (4.8%)	0.31 (0.06, 0.98)	3.66E-02	5.20E-02	2.42E-02
DPB1*04:*02*:*01*	152 (11.9%)	2 (3.2%)	0.25 (0.03, 0.94)	3.90E-02	3.81E-02	
DPB1*23:*01*:*01*	4 (0.3%)	2 (3.2%)	10.51 (0.93, 75.02)	2.83E-02		
	FDR n (%)	NT1 n (%)				
DRB3*01:*01*:*02*	18 (13.6%)	1 (1.6%)	0.1 (0, 0.69)	8.09E-03		
DRB1*03:*01*:*01*	16 (12.1%)	1 (1.6%)	0.12 (0, 0.81)	1.40E-02		
DQA1*01:*02*:*01*	55 (41.7%)	38 (61.3%)	2.22 (1.2, 4.11)	1.07E-02		
DQA1*03:02//*03:*03*:*01*	13 (9.8%)	9 (14.5%)	1.55 (0.63, 3.86)	3.39E-01	3.26E-02	
DQB1*02:*01*:*01*	16 (12.1%)	1 (1.6%)	0.12 (0, 0.81)	1.40E-02		

Although not revealed in the RPE analysis, it was observed in the haplo.score analysis that DRB3*01:*01*:*02* and non-amplified *HLA*-*DRB3*, *DRB4*, and *DRB5* were negatively associated with NT1 patients compared to GP (S2 Table). HLA-DRB5*01:*01*:*01* was observed with increased frequency for patients compared to FDR (S6 Table).

### *HLA*-*DRB1* alleles

Twenty different alleles of *HLA*-*DRB1* were found at a frequency of >1% among patients or GP (S2 Table); 24 different *HLA*-*DRB1* alleles were found only among GP at a frequency lower than 1% (S3 Table). The RPE analysis with sequential elimination of each *HLA-DRB1* allele with the strongest association showed that DRB1*15:*01*:*01*, DRB1*04:*01*:*01*, and DRB1*13:*02*:*01* were associated with NT1 (Table 1 and S4 Table). HLA-DRB1*03:*01*:*01* was found at a lower frequency among NT1 patients than among FDR (Table 1 and S5 Table).

Although not revealed in the RPE analysis, it was observed in the haplo.score analysis that *HLA*-DRB1*01:*01*:*01*, DRB1*03:*01*:*01*, and DRB1*13:*01*:*01* were negatively associated with NT1 compared to GP (S2 Table). DRB1*15:*01*:*01* was observed to be associated with NT1 compared to FDR (S6 Table).

### *HLA*-*DQA1* alleles

Thirteen different alleles of *HLA*-*DQA1* were found among NT1 patients or GP in a frequency >1% (S2 Table). Also, ten different *HLA*-*DQA1* alleles were found only among GP in a frequency lower than 1% (S3 Table).

RPEs analysis revealed that DQA1*01:*02*:*01* and DQA1*03:02//*03:*03*:*01* were associated with NT1 compared to GP (Table 1 and S4 Table) and FDR (Table 1 and S5 Table).

Not revealed by RPE, it was observed in the haplo.score analysis that DQA1*05:*01*:*01* were negatively associated with NT1 patients compared to GP (S2 Table) and FDR (S6 Table). Lower allele frequency among patients compared to GP were observed for DQA1*01:*01*:*01*, DQA1*01:*03*:*01*, and DQA1*03:*01*:*01* (S2 Table).

### *HLA*-*DQB1* alleles

Twelve different alleles of *HLA*-*DQB1* were found among patients or GP in a frequency of at least >1% (S2 Table). Twelve additional alleles were found only among GP and at a frequency lower than 1% (S3 Table). RPE analysis with sequential elimination of each *HLA-DQB1* allele with the strongest association showed that DQB1*06:*02*:*01*, DQB1*03:*01*:*01* and DQB1*06:*04*:*01* were associated with NT1 (Table 1 and S4 Table). DQB1*02:01:01 were found at a lower frequency among NT1 patients than among FDRs (Table 1 and S5 Table).

Although not detected in the RPE analysis, it was noted that *HLA*-DQB1*02:*01*:*01*, DQB1*05:*01*:*01* and DQB1*06:*03*:*01* were negatively associated with NT1 compared to GP (S2 Table). *HLA*-DQB1*06:*02*:*01* was observed to be associated with NT1 patients compared to FDR (S6 Table).

### *HLA*-*DPA1* and *DPB1* alleles

Six different alleles of *HLA*-*DPA1* and fifteen different alleles of *DPB1* were found among patients or GP in a frequency >1% (S2 Table). A total of six different alleles of *HLA*-*DPA1* and eleven of *DPB1* were found only among GP at a frequency lower than 1% (S3 Table).

RPE analysis revealed that DPA1*02:*06*:*01* and DPB1*23:*01*:*01* were associated with NT1 compared to GP. DPB1*02:*01*:*02* and DPB1*04:*02*:*01* were less common in patients compared to GP (Table 1 and S4 Table).

### *HLA*-*DRB3*, *DRB4*, and *DRB5-DRB1* haplotypes

A total of 24 different haplotypes were found among either patients or GP in a frequency of >1% (S7 Table); 43 different haplotypes were found only among GP and in lower frequency than 1% (S8 Table). In the RPE analysis, DRB5*01:*01*:01-DRB1*15:*01*:*01*, DRB4*01:*03*:01-DRB1*04:*01*:*01* and DRB3*03:*01*:01-DRB1*13:*02*:*01* were found in higher frequency among patients as compared to GP (Table 2 and S9 Table). Although not detected in the RPE analysis, it was noted that *HLA*-DRB3*01:*01*:02-DRB1*03:*01*:*01* and non-amplified *HLA*-*DRB3*, *DRB4*, *DRB5*-DRB1*01:*01*:*01* were found at lower frequencies in patients compared to GP (S7 Table).

**Table 2:** *HLA-DRB3*, *DRB4*, and *DRB5-DRB1-DQA1-DQB1* haplotype frequencies among NT1 patients (n = 31) and population controls (n = 636).

HLA-*DRB3*, *DRB4*, *DRB5- DRB1* and *HLA-DQA1-DQB1* haplotypes	Controls n (%)	NT1 n (%)	OR (95% CI)	p-value	p-value	p-value	p-value
DRB5*01:*01*:01-DRB1*15:*01*:*01*	201 (15.8%)	34 (54.8%)	6.47 (3.84, 10.91)	3.31E-15			
DRB4*01:*03*:01-DRB1*04:*01*:*01*	123 (9.7%)	7 (11.3%)	1.19 (0.53, 2.67)	6.74E-01	2.88E-02		
DRB3*03:*01*:01-DRB1*13:*02*:*01*	55 (4.3%)	4 (6.5%)	1.53 (0.39, 4.35)	3.49E-01	5.84E-02	3.40E-02	
DQA1*01:*02*:01-DQB1*06:*02*:*01*	200 (15.7%)	34 (54.8%)	6.51 (3.86, 10.97)	2.62E-15			
DQA1*03:02//DQA1*03:*03*:01-DQB1*03:*01*:*01*	56 (4.4%)	7 (11.3%)	2.76 (1.2, 6.34)	1.25E-02	8.75E-06		
DQA1*01:*02*:01-DQB1*06:*04*:*01*	52 (4.1%)	4 (6.5%)	1.62 (0.41, 4.63)	3.26E-01	4.95E-02	2.30E-02	
**HLA-*DRB3*, *DRB4*, *DRB5-DRB1-DQA1-DQB1* haplotypes**							
DRB5*01:*01*:01-*15:*01*:01-*01:*02*:01-*06:*02*:*01*	194 (15.3%)	34 (54.8%)	6.75 (4, 11.38)	6.17E-16			
DRB4*01:*03*:01-*04:*01*:01-*03:*02//03*:*03*:01-*03:*01*:*01*	39 (3.1%)	5 (8.1%)	2.77 (0.82, 7.42)	4.94E-02	3.93E-03		
DRB3*03:*01*:01-*13:*02*:01-*01:*02*:01-*06:*04*:*01*	51 (4%)	4 (6.5%)	1.65 (0.42, 4.72)	3.19E-01	4.60E-02	2.73E-02	
DRB4*01:*03*:01-*07:*01*:01-*02:01//*02:*01*:01-*02:*02*:*01*	41 (3.2%)	3 (4.8%)	1.53 (0.29, 5.01)	4.55E-01	9.63E-02	6.59E-02	4.62E-02

### *HLA-DQA1-DQB1* haplotypes

A total of seventeen different *HLA*-*DQA1-DQB1* haplotypes were found among patients or GP (>1%) (S7 Table); 32 haplotypes were associated only to GP and in a frequency lower than 1% (S8 Table). In the RPE analysis, DQA1*01:*02*:01-DQB1*06:*02*:*01*, DQA1*03:02//*03:*03*:01-DQB1*03:*01*:*01*, and DQA1*01:02:01-DQB1*06:04:01 were associated with NT1 compared to GP (Table 2 and S9 Table). Although not revealed in the RPE analysis, it was noted that DQA1*01:*01*:01-DQB1*05:*01*:*01*, DQA1*01:*03*:01-DQB1*06:*03*:*01*, DQA1*03:*01*:01-DQB1*03:*01*:*01* and DQA1*05:*01*:01-DQB1*02:*01*:*01* were found at a lower frequency among patients than among GP (S7 Table).

### *HLA*-*DRB3*, *DRB4*, and *DRB5-DRB1- DQA1-DQB1* haplotypes

The high-resolution *HLA* typing revealed a total of 26 different *HLA*-*DRB3*, *DRB4*, and *DRB5-DRB1-DQA1-DQB1* haplotypes among NT1 patients and GP (>1%) (S10 Table); in GP, 88 haplotypes at a frequency lower than 1% were found (S11 Table).

The RPE analysis revealed that *HLA*-DRB5*01:*01*:01-DRB1*15:*01*:01-DQA1*01:*02*:01-DQB1*06:*02*:*01*, HLA-DRB4*01:*03*:01-DRB1*04:*01*:01-DQA1*03:02//*03:*03*:01-DQB1*03:*01*:*01*, DRB3*03:*01*:01-*13:*02*:01-*01:*02*:01-*06:*04*:*01* and DRB4*01:*03*:01-*07:*01*:01-*02:01//*02:*01*:01-*02:*02*:*01* were associated with NT1 (Table 2 and S12 Table).

Although not detected in the RPE analysis, two haplotypes were less common among patients in *HLA*-DRB3*01:*01*:02-DRB1*03:*01*:01-DQA1*05:*01*:01-DQB1*02:*01*:*01* and non-amplified *HLA*-*DRB3*, *DRB4*, DRB5-DRB1*01:*01*:01-DQA1*01:*01*:01-DQB1*05:*01*:*01* (S10 Table). The *HLA*-DRB5*01:*01*:01-DRB1*15:*01*:01-DQA1*01:*02*:01-DQB1*06:*02*:*01* extended haplotype was increased among NT1 patients (34/62 haplotypes, 54.8%) compared to GP (194/1272 haplotypes, 15.3%). This extended *HLA* haplotype was indeed found in 30/31 patients (96.8%) and in 178/636 GP (28.0%).

### *HLA*-*DRB3*, *DRB4*, and *DRB5-DRB1- DQA1-DQB1* genotypes

Extended *DRB3*, *DRB4*, and *DRB5-DRB1-DQA1-DQB1* genotypes were compared in NT1 (n = 30) and GP (n = 178) subjects who had the DRB5*01:*01*:01-DRB1*15:*01*:01-DQA1*01:02-DQB1*06:*02* haplotype (S13 and S14 Tables). The DRB4*01:*03*:01-DRB1*04:*01*:01-DQA1*03:02//*03:*03*:01-DQB1*03:*01*:*01* haplotype was associated with NT1, while homozygosity for DRB1*15:*01*:01-DQB1*06:*02*:*01* was not (Table 3 and S13 and S14 Tables).

**Table 3:** *HLA-DRB3*, *DRB4*, and *DRB5-DRB1-DQA1-DQB1* genotype frequencies among NT1 patients (n = 31) and population controls (n = 636).

Genotypes	Controls n (%)	NT1 n (%)	OR (95% CI)	p-value	p-value	p-value	p-value	p-value	p-value
DRB5*01:*01*:01-*15:*01*:01-*01:*02*:01-*06:*02*:01-DRB4*01:*03*:01-*04:*01*:01-*03:*02//03*:*03*:01-*03:*01*:*01*	6 (0.9%)	5 (16.1%)	19.89 (4.5, 84.19)	5.93E-05					
DRB5*01:*01*:01-*15:*01*:01-*01:*02*:01-*06:*02*:01-DRB5*01:*01*:01-*15:*01*:01-*01:*02*:01-*06:*02*:*01*	16 (2.5%)	4 (12.9%)	5.71 (1.3, 19.4)	1.10E-02	6.11E-03				
DRB5*01:*01*:01-*15:*01*:01-*01:*02*:01-*06:*02*:01-DRB3*03:*01*:01-*13:*02*:01-*01:*02*:01-*06:*04*:*01*	10 (1.6%)	3 (9.7%)	6.66 (1.12, 27.89)	1.90E-02	1.22E-02	8.22E-03			
DRB5*01:*01*:01-*15:*01*:01-*01:*02*:01-*06:*02*:01-DRBX*null-*08:*01*:01-*04:*01*:01-*04:*02*:*01*	6 (0.9%)	2 (6.5%)	7.19 (0.68, 42.55)	4.92E-02	3.65E-02	2.82E-02	2.21E-02		
DRB5*01:*01*:01-*15:*01*:01-*01:*02*:01-*06:*02*:01-DRB3*02:*02*:01-*12:*01*:01-*05:*05*:01-*03:*01*:*01*	7 (1.1%)	2 (6.5%)	6.16 (0.6, 34.34)	6.14E-02	4.58E-02	3.55E-02	2.79E-02	2.31E-02	
DRB5*01:*01*:01-*15:*01*:01-*01:*02*:01-*06:*02*:01-DRB4*01:*03*:01-*07:*01*:01-*02:*01//02*:*01*:01-*02:*02*:*01*	8 (1.3%)	2 (6.5%)	5.39 (0.53, 28.73)	7.46E-02	5.59E-02	4.35E-02	3.43E-02	2.84E-02	2.30E-02
*HLA-DRB3*, *DRB4*, *DRB5-DRB1-DQA1-DQB1* haplotypes among HLA-DRB5*01:*01*:01-DRB1*15:*01*:01-DQA1*01:*02*:01-DQB1*06:*02*:*01* heterozygotes									
DRB4*01:*03*:01-*04:*01*:01-*03:02//*03:*03*:01-*03:*01*:*01*	6 (4%)	5 (16.7%)	4.77 (1.07, 20.41)	2.02E-02					

In total, 346 different *DRB3*, *DRB4*, and *DRB5-DRB1-DQA1-DQB1* genotypes were found among 667 subjects (31 NT1 patients and 636 GP); 25 genotypes were found among either patients or GP in a frequency of >1% (S15 and S16 Tables). Six extended genotypes were associated to NT1, all had one *HLA*-DRB5*01:*01*:01-DRB1*15:*01*:01-DQA1*01:*02*:01-DQB1*06:*02*:*01* extended haplotype and were paired with either DRB4*01:*03*:01-DRB1*04:*01*:01-DQA1*03:*02//03*:*03*:01-DQB1*03:*01*:*01*, DRB5*01:*01*:01-DRB1*15:*01*:01-DQA1*01:*02*:01-DQB1*06:*02*:*01*, DRB3*03:*01*:01-DRB1*13:*02*:01-DQA1*01:*02*:01-DQB1*06:*04*:*01*, non-amplified *HLA*-*DRB3*, *DRB4*, DRB5-DRB1*08:*01*:01-DQA1*04:*01*:01-DQB1*04:*02*:*01*, DRB3*02:*02*:01-DRB1*12:*01*:01-DQA1*05:*05*:01-DQB1*03:*01*:*01* or DRB4*01:*03*:01-DRB1*07:*01*:01-DQA1*02:*01//02*:*01*:01-DQB1*02:*02*:*01* as the second haplotype (Table 3 and S15 Table).

## Discussion

High-resolution *HLA* sequencing was used to dissect the association between *HLA* and NT1 that developed in subjects vaccinated with Pandemrix. The major findings were:

1. The patients (30/31 subjects, 34/62 haplotypes) carried at least one haplotype of DRB5*01:*01*:01-DRB1*15:*01*:01-DQA1*01:*02*:01-DQB1*06:*02*:*01*.
2. Among subjects heterozygous for DRB5*01:*01*:01-DRB1*15:*01*:*01*-DQA1*01:*02*:01-DQB1*06:*02*:*01*, the only second haplotype associated with NT1 was DRB4*01:*03*:01-DRB1*04:*01*:01-DQA1*03:02//*03:*03*:01-DQB1*03:*01*:*01* while DRB5*01:01:01-DQA1*01:*02*:*01*-DRB1*15:*01*:01-DQB1*06:*02*:*01* homozygosity was not.
3. Alleles with an increased (DQA1*01:*02*:*01*, DQA1*03:02//*03:*03*:*01*) and decreased (DRB3*01:*01*:*02*, DRB1*03:*01*:*01*, DQB1*02:*01*:*01*) frequency were associated with the patients compared to their FDR.

Caucasians demonstrate a strong linkage disequilibrium with the haplotype of DRB5*01:*01*:01-DRB1*15:*01*:01-DQA1*01:*02*:01-DQB1*06:*02*:*01* and those haplotypes were found in 30/31 patients in the current study. NT1 was also positively associated with DQA1*03:02//*03:*03*:01-DQB1*03:*01*. We speculate that co-inheritance of DQA1*01:02-DQB1*06:*02* together with DQA1*03:02//*03:*03*:*01*-DQB1^*^03:*01* as secondary haplotype was a characteristic genetic predisposition for Pandemrix-induced NT1. HLA-DQB1^*^03:*01* has previously been reported to associate to NT1 if present as the second allele to DQB1*06:*02* [10–13].

The strengths of the present study were the high-resolution *HLA* typing in a group of Pandemrix-induced NT1 patients and controls both from the general population and the NT1 patients respective immediate family. Using the families, it was possible to ascertain by descent correct extended *HLA* haplotypes. Our study determined allelic frequencies for patients compared to FDR. Three alleles, DRB3*01:*01*:*02*, DRB1*03:*01*:*01*, and DQB1*02:*01*:*01* were found at a higher frequency among the FDR compared to the NT1 probands and could have had a protective impact in the etiological process leading to NT1. Although there are few family studies of NT1, it is generally accepted that the disease is non-hereditary. The discordance found among twins suggests other factors that might influence the etiology and pathogenesis of this disease in genetically predisposed individuals [31, 32].

Non-NT1-associated alleles for DQB1 (DQB1*02:*01*:*01*, DQB1*05:*01*:*01*, DQB1*06:*03*:*01*) described in large multi-national non-vaccine related cohorts [5] and among Pandemrix-NT1 patients (DQB1*05:*01*, DQB1*06:*03* and DQB1*06:*09*) [24] were mostly confirmed in our study.

To our knowledge, this is the first study examining the relationships between NT1 and *HLA*-*DRB3*, *DRB4*, and *DRB5*. Our study found NT1 to be positively associated with DRB5*01:*01*:*01*, DRB4*01:*03*:*01*, and DRB3*03:*01*:*01*. *HLA-DRB3*, *DRB4*, and *DRB5* genetics have been suggested to have a role in the autoimmune process of type 1 diabetes [27].

HLA-DPA1^*^01:03-DPB1^*^04:*02* and *HLA*-DPA1^*^01:03-DPB1^*^04:*01* were reported to be negatively associated with NT1 [15]. In our Swedish patients, we were able to confirm the decreased frequency of DPB1*04:*02*:*01* among the NT1 patients. In addition, we reported NT1 to be negatively associated with DPB1*02:*01*:*02*. The previous [15] and the present report are of interest since *HLA-DP* associations are suggested to affect the antibody response to influenza vaccination [33]. In addition, *DPB1* alleles were related to multiple sclerosis [34], a disease sharing the DRB1*15:*01*:01-DQB1*06:*02*:*01* association with NT1.

The current study presents what could be interpreted as a seemingly high number of p-values. However, it should be noted first that comparisons of *HLA* alleles are not independent of each other. Second, results and p-value of a single allele is replicated in the haplotype and often also in the extended haplotype. For the RPE analysis, the limitation of the small sample size is recognized. It is noted that these results are based on a small sample size and need to be verified independently. Limitations of this study include the lack of follow-up from NT1 genetics to clinical diagnosis and disease severity. NT1 is a complex disease as symptoms, and response to treatments varies in unpredictable ways. *HLA*-*DQA1-DQB1* haplotype preferences have been addressed in previous reports [14], but further extension into extended *HLA* haplotypes and genotypes is largely an unexplored field as spanning the *HLA* complex from the *DRB3*, *DRB4*, and *DRB5* to the *DPB1* may have implications to individual patients. As an example, *HLA* genetics in type 1 diabetes affects the preference of the first autoantibody (IAA or GADA) and has been proposed to be linked to different triggering mechanisms, although both eventually cause beta cell destruction and diabetes [35, 36].

In conclusion, high-resolution *HLA* class II sequencing demonstrated novel insights into the genetic predisposition to Pandemrix-induced NT1. The finding that the majority of NT1 patients following Pandemrix-vaccination are heterozygous for DQA1*01:*02*:01-DQB1*06:*02*:*01* and with DQA1*03:02//*03:*03*:01-DQB1*03:*01*:*01* as the most common second haplotype suggests that subjects with these *HLA* combinations have an increased genetic risk for Pandemrix-induced narcolepsy type 1.

## Supporting information

S1 Table Schematic illustration of the assembly process used to create extended haplotypes. (PDF)

S2 Table Haplo.score analysis of estimated allelic frequencies of *HLA-DRB3*, *DRB4*, *DRB5*, *-DRB1*, *-DQA1*, *-DQB1*, *-DPA1*, and *-DPB1* among NT1 patients and general population controls. (XLSX)

S3 Table Haplo.score analysis of estimated allelic frequencies of rare (< 1%) *HLA-DRB3*, *DRB4*, *DRB5*,—*DRB1*, *-DQA1*, *-DQB1*, *-DPA1*, and *-DPB1* among NT1 patients and general population controls. (XLSX)

S4 Table RPE analysis of estimated allelic frequencies of *HLA-DRB3*, *DRB4*, *DRB5*, *-DRB1*, *-DQA1*, *-DQB1*, *-DPA1*, and *-DPB1* among NT1 patients and general population controls. (XLSX)

S5 Table RPE analysis of estimated allelic frequencies of *HLA-DRB3*, *DRB4*, *DRB5*, *-DRB1*, *-DQA1*, *-DQB1*, *-DPA1*, and *-DPB1* among NT1 patients and FDR. (XLSX)

S6 Table Haplo.score analysis of estimated allelic frequencies of *HLA-DRB3*, *DRB4*, *DRB5*, *-DRB1*, *-DQA1*, *-DQB1*, *-DPA1*, and *-DPB1* among NT1 patients and FDR. (XLSX)

S7 Table Haplo.score analysis of estimated frequencies of *HLA-DR* and *HLA-DQ* haplotypes among NT1 patients and population controls. (XLSX)

S8 Table Haplo.score analysis of estimated frequencies of rare (< 1%) *HLA-DR* and *HLA-DQ* haplotypes among NT1 patients and population controls. (XLSX)

S9 Table RPE analysis of estimated frequencies of *HLA-DR* and *HLA-DQ* haplotypes among NT1 patients and population controls. (XLSX)

S10 Table Haplo.score analysis of estimated frequencies of *HLA-DRB3*, *DRB4*, and *DRB5-DRB1-DQA1-DQB1* haplotypes among NT1 patients and population controls. (XLSX)

S11 Table Haplo.score analysis of estimated frequencies of rare (< 1%) *HLA-DRB3*, *DRB4*, and *DRB5-DRB1-DQA1-DQB1* haplotypes among NT1 patients and population controls. (XLSX)

S12 Table RPE analysis of the estimated frequencies of *HLA-DRB3*, *DRB4*, and *DRB5-DRB1-DQA1-DQB1* haplotypes among NT1 patients and population controls. (XLSX)

S13 Table RPE analysis of the estimated frequencies of second *HLA-DRB3*, *DRB4*, and *DRB5-DRB1-DQA1-DQB1* haplotypes among DRB5*01:*01*:01-*15:*01*:01-*01:*02*:01-*06:*02*:*01* heterozygotes. (XLSX)

S14 Table Haplo.score analysis of the estimated frequencies of second *HLA-DRB3*, *DRB4*, and *DRB5-DRB1-DQA1-DQB1* haplotypes among DRB5*01:*01*:01-*15:*01*:01-*01:*02*:01-*06:*02*:*01* heterozygotes. (XLSX)

S15 Table RPE analysis of the estimated frequencies of *HLA-DRB3*, *DRB4*, and *DRB5-DRB1-DQA1-DQB1* genotypes among NT1 patients and population controls. (XLSX)

S16 Table Haplo.score analysis of the estimated frequencies of *HLA-DRB3*, *DRB4*, and *DRB5-DRB1-DQA1-DQB1* genotypes among NT1 patients and population controls. (XLSX)
