# Supplementary material for: HLA high-resolution typing by next-generation sequencing in Pandemrix-induced narcolepsy
Source: PLoS One. 2019 Oct 2;14(10):e0222882. doi: 10.1371/journal.pone.0222882 (PMC6774514; doi:10.1371/journal.pone.0222882)
Supplement: S1 Table — (PDF) [file pone.0222882.s001.pdf]

1. Identification of homozygous individuals

DRB3, DRB4, DRB5	DRB1	DQA1	DQB1
DRB5*01:01:01	*15:01:01	*01:02:01	*06:02:01
DRB5*01:01:01	*15:01:01	*01:02:01	*06:02:01
DRB5*01:01:01	*15:01:01	*01:02:01	*06:02:01
DRB5*01:01:01	*15:01:01	*01:02:01	*06:02:01
DRB4*01:03:01	*07:01:01	*02:01//02:01:01	*02:02:01
DRB4*01:03:01	*07:01:01	*02:01//02:01:01	*02:02:01

2. Identification of heterozygous *DRB5*01:01:01-DRB1*15:01:01-DQA1*01:02:01-DQB1*06:02:01* individuals

ID	Hap	DRB3, DRB4, DRB5	DRB1	DQA1	DQB1
1	1	DRB5*01:01:01	*03:01:01	*01:02:01	*02:01:01
	2	DRB3*01:01:02	*15:01:01	*05:01:01	*06:02:01
2	1	DRB5*01:01:01	*15:01:01	*05:01:01	*02:01:01
	2	DRB3*01:01:02	*03:01:01	*01:02:01	*06:02:01
1	1	DRB5*01:01:01	*15:01:01	*01:02:01	*06:02:01
	2	DRB3*01:01:02	*03:01:01	*05:01:01	*02:01:01
2	1	DRB5*01:01:01	*15:01:01	*01:02:01	*06:02:01
	2	DRB3*01:01:02	*03:01:01	*05:01:01	*02:01:01
		DRB3, DRB4, DRB5	DRB1	DQA1	DQB1
3	1	DRBX*null	*03:01:01	*05:01:01	*05:01:01
	2	DRB3*01:01:02	*01:01:01	*01:01:01	*02:01:01
4	1	DRB3*01:01:02	*01:01:01	*05:01:01	*05:01:01
	2	DRBX*null	*03:01:01	*01:01:01	*02:01:01
3	1	DRB3*01:01:02	*03:01:01	*05:01:01	*02:01:01
	2	DRBX*null	*01:01:01	*01:01:01	*05:01:01
4	1	DRB3*01:01:02	*03:01:01	*05:01:01	*02:01:01
	2	DRBX*null	*01:01:01	*01:01:01	*05:01:01

3. A total of 16 different extended haplotype variants were identified

	DRB3, DRB4, DRB5	DRB1	DQA1	DQB1	n	DRB3, DRB4, DRB5	DRB1	DQA1	DQB1	n	DRB3, DRB4, DRB5	DRB1	DQA1	DQB1	n
1	DRB5*01:01:01	*15:01:01	*01:02:01	*06:02:01	194	DRB5*01:01:01	*15:01:01	*01:02:01	*06:14:01	1	DRBX*null	*15:01:01	*01:02:01	*06:02:01	1
						DRB5*01:01:01	*15:01:01	*01:02:01	*05:01:01	1	DRB5*01:01:01	*16:09	*01:02:01	*06:02:01	1
						DRB5*01:01:01	*15:01:01	*01:02:02	*05:02:01	1	DRB5*01:01:01	*15:03:01	*01:02:01	*06:02:01	2
											DRB5*01:01:01	*15:01:01	*01:02:01	*06:03:01	4
2	DRB3*01:01:02	*03:01:01	*05:01:01	*02:01:01	125	DRB3*02:02:01	*03:01:01	*05:01:01	*02:01:08	1					
	DRB3*02:02:01	*03:01:01	*05:01:01	*02:01:01	19										
	DRB3*01:01:02	*03:01:01	*05:01:01	*02:01:08	8										
3	DRB4*01:03:01	*04:01:01	*03:01:01	*03:02:01	70	DRB4*01:03:01	*04:01:01	*03:02//03:03:01	*03:01:03	1	DRB4*01:03:02	*04:07:01	*03:01:01	*03:02:01	1
	DRB4*01:03:01	*04:01:01	*03:02//03:03:01	*03:01:01	39	DRB4*01:03:01	*04:01:01	*05:05:01	*03:01:01	1	DRB4*01:03:02	*04:07:01	*03:01:01	*03:02:01	1
	DRB4*01:03:01	*04:01:01	*03:01:01	*03:01:01	6	DRB4*01:01:01// DRB4*01:01:01:01	*04:01:01	*03:02//03:03:01	*03:01:01	1	DRB4*01:03:01	*04:08:01	*03:01:01	*03:02:01	1
	DRB4*01:03:01	*04:01:01	*03:02//03:03:01	*03:02:01	6	DRB4*01:02	*04:01:01	*03:02//03:03:01	*03:01:01	2	DRB4*01:03:01	*04:08:01	*03:02//03:03	*03:01:01	4
	DRB4*01:03:01	*04:07:01	*03:02//03:03:01	*03:01:01	9	DRB4*01:03:01	*04:05:01	*03:02//03:03:01	*03:02:01	3	DRB4*01:03:01	*04:08:01	*03:02//03:03	*03:04// *03:04:01	3
	DRB4*01:03:01	*09:01:02	*03:02//03:03:01	*03:03:02	8	DRB4*01:03:01	*04:05:04	*03:01:01	*04:02:01	1	DRB4*01:03:01	*04:10:01	*03:02//03:03	*04:02:01	1
						DRB4*01:03:01	*04:06:01	*03:02//03:03:01	*04:02:01	1	DRB4*01:03:01	*04:13	*03:02//03:03	*03:01:01	1
						DRB4*01:03:01	*04:07:01	*03:01:01	*03:02:01	1	DRB4*01:03:01	*09:01:02	*03:01:01	*03:03:02	2
											DRB4*01:03:02	*09:01:02	*03:02//03:03	*03:03:02	2
4	DRBX*null	*01:01:01	*01:01:01	*05:01:01	113	DRBX*null	*01:01:01	*01:02:01	*05:04	3					
	DRBX*null	*01:02:01	*01:01:01	*05:01:01	13	DRB3*01	*01:01:01	*01:01:01	*05:01:01	1					
	DRBX*null	*01:03//*01:03:01	*01:01:01	*05:01:01	9	DRBX*null	*01:03//*01:03:01	*05:05:01	*03:01:01	2					
	DRBX*null	*10:01:01	*01:01:01	*05:01:01	7	DRBX*null	*10:01:01	*01:05:01	*05:01:01	1					
5	DRB4*01:01:01// DRB4*01:01:01:01	*07:01:01	*02:01//02:01:01	*02:02:01	37	DRB4*01:03:01:02N	*07:01:01	*02:01//02:01:01	*03:03:02	5					
	DRB4*01:03:01	*07:01:01	*02:01//02:01:01	*02:02:01	41										
	DRB4*01:03:01	*07:01:01	*02:01//02:01:01	*03:03:02	31										
6	DRB3*01:01:02	*13:01:01	*01:03:01	*06:03:01	54	DRB3*01	*13:01:01	*01:03:01	*06:03:01	1					
	DRB3*02:02:01	*13:01:01	*01:03:01	*06:03:01	36	DRB3*01:01:02	*13:01:01	*01:10	*06:03:01	1					
7	DRB3*02:02:01	*11:01:01	*05:05:01	*03:01:01	57	DRB3*02:02:01	*11:01:01	*05:03	*03:01:01	2	DRB3*02:02:01	*11:04:01	*01:03:01	*06:03:01	1
	DRB3*02:02:01	*11:03//11:03:01	*05:05:01	*03:01:01	6	DRB3*02:02:01	*11:01:01	*05:09	*03:01:01	1	DRB3*02:02:01	*11:04:01	*05:03	*03:01:01	1
	DRB3*02:02:01	*11:04:01	*05:05:01	*03:01:01	16	DRB3*02:02:01	*11:01:01	*05:10	*03:01:01	1	DRB3*02:02:01	*11:84:01	*05:05:01	*03:01:01	1
	DRB3*02:02:01	*12:01:01	*05:05:01	*03:01:01	27	DRB3*01:01:02	*11:01:01	*05:05:01	*03:01:01	2	DRB3*02:02:01	*12:01:01	*05:05:01	*03:02:01	1
						DRB3*02:02:01	*11:01:02	*01:02:01	*06:02:01	1	DRB3*02:02:01	*13:05:01	*05:05:01	*03:02:01	1
						DRB3*02:02:01	*11:01:02	*05:05:01	*03:19:01//03:19:01	1	DRB3*02:02:01	*08:04:01	*05:05:01	*03:01:01	1
						DRB3*03:01:01	*11:01:02	*01:02:01	*06:02:01	1	DRBX*null	*08:04:01	*05:05:01	*03:19:01//03:19:01	1
						DRB3*02:02:01	*11:02:01	*05:05:01	*03:01:01	3	DRB3*01:01:02	*14:02	*05:03	*03:01:01	3
						DRB3*02:02:01	*11:02:01	*05:05:01	*03:19:01//03:19:01	1	DRB3*01:01:02	*14:12:01	*05:03	*03:01:01	1
8	DRB4*01:03:01	*04:04:01	*03:01:01	*03:02:01	59	DRB4*01:03:02	*04:04:01	*03:01:01	*03:02:01	1	DRB4*01:03:01	*04:03:01	*03:01:01	*03:05:01	2
	DRB4*01:03:01	*04:02:01	*03:01:01	*03:02:01	6	DRB4*01:03:01:02N	*04:02:01	*03:01:01	*03:02:01	1	DRB4*01:03:01	*04:03:01	*03:01:01	*03:04// *03:04:01	1
	DRB4*01:03:01	*04:03:01	*03:01:01	*03:02:01	8						DRBX*null	*04:04:01	*03:01:01	*03:02:01	1
9	DRB3*03:01:01	*13:02:01	*01:02:01	*06:04:01	51	DRB3*03:01:01	*13:02:01	*01:02:01	*06:09 // *06:09:01	2	DRB3*03:01:01	*13:02:01	*01:02:01	*05:01:01	2
						DRB3*02:38	*13:02:01	*01:02:01	*06:04:01	1					
10	DRBX*null	*08:01:01	*04:01:01	*04:02:01	47	DRBX*null	*08:01:01	*04:02	*04:02:01	3	DRBX*null	*08:01:01	*04:01:01	*03:03:02	1
						DRB3*02:02:01	*08:01:01	*04:01:01	*04:02:01	1	DRBX*null	*08:01:01	*04:04	*04:02:01	1
											DRBX*null	*08:02:01	*04:01:01	*04:02:01	3
11	DRB3*02:02:01	*14:54:01	*01:01:01	*05:03:01	12	DRB3*02:02:01	*14:01:01	*01:01:01	*05:03:01	1	DRB3*02:02:01	*14:54:01	*01:07Q	*05:03:01	1
	DRB3*02:02:01	*14:54:01	*01:04:01	*05:03:01	7	DRB3*02:24	*14:01:01	*01:01:01	*05:03:01	3	DRB3*02:02:01	*14:54:01	*01:07	*05:03:01	1
						DRB3*02:24	*14:01:01	*01:04:01	*05:03:01	1	DRBX*null	*14:54:01	*01:01:01	*05:03:01	1
						DRB3*02:02:01	*14:04//*14:04:01	*01:01:01	*05:03:01	1					
12	DRB5*02:02// DRB5*02:02:01	*16:01:01	*01:02:02	*05:02:01	14	DRB5*02:02// DRB5*02:02:01	*16:01:01	*01:02:02	*06:02:01	1	DRB5*02:02// DRB5*02:02:01	*16:01:01	*01:02:02	*06:03:01	1
13	DRBX*null	*08:03:02	*06:01:01	*03:01:01	4										
	DRBX*null	*08:03:02	*01:03:01	*06:01:01	3										
14	DRB5*01:02	*15:02:01	*01:03:01	*06:01:01	6	DRB5*01:08	*15:02:01	*01:01:01	*05:01:01	1	DRB5*01:01:01	*15:02:01	*01:01:01	*05:01:01	1
						DRB5*01:02	*15:02:01	*01:01:01	*05:01:24	1					
15	DRB3*01:01:02	*13:03:01	*05:05:01	*03:01:01	9	DRB3*01:01:02	*13:03:01	*05:05:01	*03:09	1					
16	DRB3*03:01:03	*12:02:01	*01:02:02	*05:02:01	1										
	DRB3*03:01:03	*12:02:01	*06:01:01	*03:01:01	2										

4. Conflicting results were found for two individuals. In both cases the two extended haplotypes were matched to the same HLA *DRB3, DRB4, DRB5* allele. This was solved by assigning the matched *DRB3, DRB4, DRB5* to the more common haplotype.

DRB3, DRB4, DRB5	DRB1	DQA1	DQB1
DRB3*01:01:02	*13:01:01	*01:03:01	*06:03:01
DRB3*03:01:01	*11:01:02	*01:02:01	*06:02:01
DRB4*01:03:01	*07:01:01	*02:01//02:01	*02:02:01
DRBX*null	*04:04:01	*03:01:01	*03:02:01
